# From transcriptional landscapes to the identification of biomarkers for robustness

**DOI:** 10.1186/1475-2859-10-S1-S9

**Published:** 2011-08-30

**Authors:** Tjakko Abee, Michiel Wels, Mark de Been, Heidy den Besten

**Affiliations:** 1 Laboratory of Food Microbiology, Wageningen University, Wageningen, The Netherlands; 2 TI Food and Nutrition, Wageningen, The Netherlands; 3 Centre for Molecular and Biomolecular Informatics (CMBI), NCMLS, Radboud University Nijmegen Medical Centre, Nijmegen, The Netherlands; 4 NIZO food research, Ede, The Netherlands

## Abstract

The ability of microorganisms to adapt to changing environments and gain cell robustness, challenges the prediction of their history-dependent behaviour. Using our model organism *Bacillus cereus*, a notorious Gram-positive food spoilage and pathogenic spore-forming bacterium, a strategy will be described that allows for identification of biomarkers for robustness. First an overview will be presented of its two-component systems that generally include a transmembrane sensor histidine kinase and its cognate response regulator, allowing rapid and robust responses to fluctuations in the environment. The role of the multisensor hybrid kinase RsbK and the PP2C-type phosphatase RsbY system in activation of the general stress sigma factor σ^B^ is highlighted. An extensive comparative analysis of transcriptional landscapes derived from *B. cereus* exposed to mild stress conditions such as heat, acid, salt and oxidative stress, revealed that, amongst others σ^B^ regulated genes were induced in most conditions tested. The information derived from the transcriptome data was subsequently implemented in a framework for identifying and selecting cellular biomarkers at their mRNA, protein and/or activity level, for mild stressinduced microbial robustness towards lethal stresses. Exposure of unstressed and mild stress-adapted cells to subsequent lethal stress conditions (heat, acid and oxidative stress) allowed for quantification of the robustness advantage provided by mild stress pretreatment using the plate-count method. The induction levels of the selected candidate-biomarkers, σ^B^ protein, catalase activity and transcripts of certain proteases upon mild stress treatment, were significantly correlated to mild stress-induced enhanced robustness towards lethal thermal, oxidative and acid stresses, and were therefore suitable to predict these adaptive traits. Cellular biomarkers that are quantitatively correlated to adaptive behavior will facilitate our ability to predict the impact of adaptive behavior on cell robustness and will allow to control and/or exploit these adaptive traits. Extrapolation to other species and genera is discussed such as avenues towards mechanism-based design of microbial fitness and robustness.

## Introduction

*Bacillus cereus* is a member of the genus *Bacillus*, which is a highly heterogeneous group of bacteria that includes species with large variations in phenotypes, nutritional requirements and other physiological and metabolic characteristics. Within the genus *Bacillus*, *B. cereus* and its closest relatives form a highly homogeneous subdivision, which has been termed the “*B. cereus* group”. This group comprises the species *B. cereus*, *B. anthracis*, *B. thuringiensis*, *B. mycoides*, *B. pseudomycoides* and *B. weihenstephanensis*. *B. anthracis* is the etiological agent of the acute and fatal disease anthrax in mammals. It is the most notorious member of the *B. cereus* group, especially since it was used in bioterrorist attacks in the USA in 2001 [1].

Members of the *B. cereus* group are ubiquitously present in soil and can adapt to a wide range of environmental conditions [2-4]. The ecological niches and life cycles of *B. anthracis* and *B. thuringiensis* may be more specialized than those of *B. cereus*. *B. anthracis* spores are ingested by herbivores and germinate within the host to produce vegetative cells, which multiply and produce virulence factors, ultimately killing the host. Upon death, large numbers of *B. anthracis* cells are released into the soil, where they sporulate upon contact with air, thus completing the *B. anthracis* life cycle [5]. The life cycle of *B. thuringiensis* may be comparable to that of *B. anthracis*. Upon death of its insect host, *B. thuringiensis* is released into the soil where it is a ubiquitous inhabitant and where it can germinate and grow under favorable conditions. [4]. The niches and life cycle of pathogenic *B. cereus* remain more obscure. *B. cereus* spores can be found in many types of soils, sediments, dust and plants [3,4,6]. For this reason, *B. cereus* is considered to be mainly a soil inhabitant and, indeed, *B. cereus* can germinate, grow and sporulate in soil, thus demonstrating a saprophytic life cycle [7]. However, genome analyses have indicated that *B. cereus* specializes in protein metabolism, pointing towards a symbiotic or parasitic life cycle [8]. This is further supported by the fact that *B. cereus* has been isolated from guts of soil-dwelling arthropods [9] and from stool samples of healthy humans [4]. Furthermore, *B. cereus* has been isolated from surface waters, a niche that may allow this bacterium to easily enter food-processing lines [10]. Many types of food have been associated with *B. cereus* food-borne disease, including meats, vegetables, puddings, milk, rice, pasta and noodles [3,11].

*B. cereus* can cause two distinct types of food-borne disease: the diarrhoeal and the emetic type. Although both types are generally mild and self-containing, more serious and even lethal cases have been reported [12-15]. The diarrhoeal disease is often associated with protein-rich foods, such as meat, vegetables, puddings and milk products. The diarrhoeal disease is thought to be caused by vegetative cells (ingested as viable cells or spores) that produce enterotoxins in the small intestine. Typical symptoms include abdominal pains, watery diarrhoea, nausea and vomiting. The incubation time generally ranges between 8-16 hours after ingestion, while the symptoms normally last for 12-24 hours. However, longer incubation times have been observed and the symptoms can last for up to several days [3]. The emetic disease is often associated with starch-rich foods, such as fried and cooked rice, pasta and noodles. It was first identified in 1974 when *B. cereus* was linked to several outbreaks caused by eating cooked rice in the United Kingdom in the early 1970s [16]. The emetic disease is caused by the *B. cereus* emetic toxin, cereulide, which is produced in foods before ingestion. Symptoms mainly include nausea and vomiting, which occur between 30 minutes to 6 hours after ingestion and which generally last for 6-24 hours [17].

Because of the medical and economic relevance of *B. cereus* group members, a large number of genomes has been sequenced. Starting with the first publications in 2003 [8,18], the list of sequenced members now includes five *B. anthracis*, eleven *B. cereus*, three *B. thuringiensis*, one *B. weihenstephanensis*, and an additional 89 sequences are in progress or available with high coverage draft sequence (Dec 2010; http://www.ncbi.nlm.nih.gov/genomes/Iproks.cgi). These sequences provide an extensive source for the identification of signaling systems and key factors in adaptive responses towards environmental and food-relevant stresses.

## Two-component signal transduction

The ubiquitous presence and high adaptive capabilities of *B. cereus* and its closest relatives may be explained by the fact that these species harbor dedicated signaling pathways that allow rapid and robust responses to fluctuations in their environment. In bacteria, including archaebacteria, the dominant signaling pathways for monitoring environmental cues are the so-called two-component systems (TCS), which include a transmembrane sensor histidine kinase (HK) and its cognate response regulator (RR). The mode of signal transduction by TCSs involves a phosphotransfer reaction between a conserved histidine and aspartate residue located in the HK phosphotransferase and RR receiver domain, respectively. RRs generally function as transcription factors that, upon phosphorylation, bind to specific sites on the DNA to alter the expression of genes involved in adaptive responses [19]. These systems are almost ubiquitously present in bacteria [20] and analyses of whole-genome and metagenome sequences have revealed that TCS protein domains are the second most numerous Pfam domains in bacteria, exceeded only by ABC-type transporter domains [21,22]. TCSs are known to monitor a wide variety of conditions, such as nutrient deprivation, cold/heat shock and the presence of antimicrobial compounds [23-25], and they can control virtually all types of microbial behaviour, including motility and chemotaxis, sporulation, biofilm formation and quorum sensing [26-29].

**Figure 1:**
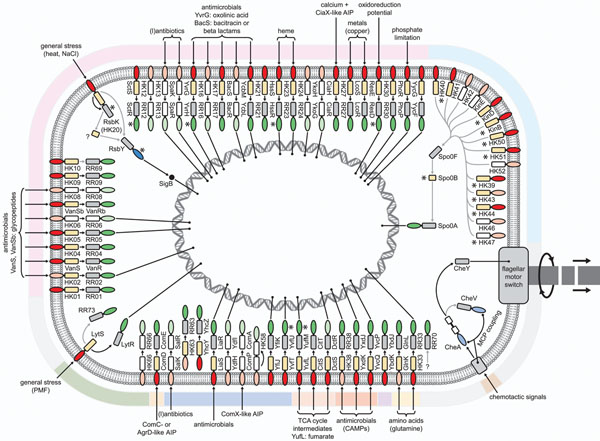
**Overview of TCSs in *B. cereus*.** HKs are indicated in red (sensory) and ecru (phosphotransferase) and RRs are indicated in grey (receiver) and green (DNA-binding domain). Blue/purple domains indicate protein-protein interaction domains. White and light (red, green, blue/purple) domains indicate the absence/truncation of the respective domain in at least two of the *B. cereus* group members analysed [30]. Coloured bars surrounding the cell represent different HK subfamilies as defined previously [74]. Incoming arrows indicate predicted or established HK-specific stimuli. Inside the cell, black and grey arrows represent predicted phosphotransfers between paired and “orphan” HKs and RRs, respectively [30]. RRs for which specific operators have been predicted are “connected” to the DNA [31]. Other connection lines illustrate protein-protein interactions. TCSs/proteins that have been experimentally studied are highlighted with asterisks. These include: RsbK [32], RsbY [33], SctRS [75], YvrHG [76,77], HssRS [78], ResDE [79-82], several sporulation HKs [83-85], Spo0B [86], YufLM [77] and YvfTU [87]. Abbreviations: AIP, auto-inducing peptide; MCP, methyl-accepting chemotaxis protein receptor; CAMP, cationic antimicrobial peptide; TCA, tricarboxylic acid; PMF, proton-motive force. For TCS numbering, see de Been et al. [30].

Recent studies by de Been at al. [30-32] and others (Fig. 1) have provided a better understanding of the contribution of TCS to the omnipresence and high adaptive capabilities of *B. cereus*, which contribute to the problematic nature of this organism to food processing industries. In addition to a large number of HKs involved in the initiation of sporulation, predicted and established biological roles for other *B. cereus* group TCS proteins include roles in biofilm formation, host-microbe interactions, chemotaxis, nutrient uptake, antibiotic resistance, and many more (Fig. 1 and references therein). As compared to other low-GC Gram-positives, the number of TCS proteins in members of the *B. cereus* group, each having around 40 HK-RR gene pairs and additional HK and RR genes not encoded in pairs (’ ’orphans’’), appears to be relatively large. This large number probably directly relates to the relatively large genome sizes of *B. cereus* group members, as larger genomes tend to encode disproportionately more signal transduction proteins. Similarly, free-living bacteria tend to encode more signal transduction proteins than highly specialized pathogenic bacteria, which generally have smaller genomes [20]. Considering this, it was of special interest to find that *B. anthracis* contains a large number of truncated, putatively non-functional, HK and RR genes and completely lacks specific HK and RR genes. A possible scenario is that specialization of *B. anthracis* as a pathogen has reduced the range of environmental stimuli it encounters, ultimately resulting in the evolutionary disposal of specific TCS genes [30].

Recent studies have identified the complex, multisensor hybrid HK RsbK and the PP2C-type phosphatase-containing RR RsbY (Fig. 1), to be involved in controlling σ^B^ activity [32,33]. In several Gram-positive bacteria, including the model Gram-positive *Bacillus subtilis*, the alternative sigma factor σ^B^ plays a prominent role in redirecting gene expression under stress conditions [34]. In *B. cereus*, activation of σ^B^ is also induced under a variety of stress conditions, most notably heat stress, and *B. cereus sigB and rsbK* deletion strains displayed an impaired heat adaptive response [32,35]. In *B. anthracis*, σ^B^ also plays a role in heat stress and a *B. anthracissigB* deletion strain was slightly affected in its virulence [36].

Further experiments in combination with *in silico* analysis [32,37] revealed that σ^B^ activation in *B. cereus* significantly differs from known σ^B^ activation pathways in other low-GC Gram-positives. In *B. subtilis*, but most likely also in many of its closest relatives, a massive protein complex called the stressosome integrates multiple environmental stress signals to orchestrate dephosphorylation of RsbV and the eventual release and activation of σ^B^[38,39]. In addition, *B. subtilis* even uses a second pathway, which monitors energy stress, to regulate σ^B^ activity [40]. Both of the above pathways are absent in members of the *B. cereus* group, where RsbY is the sole PP2C-type phosphatase for the control of σ^B^ activity [41]. RsbY most likely receives its input signals from the hybrid HK RsbK, either indirectly via an extended phosphorelay or directly. The domain architecture of the unique sensory protein RsbK suggests that in members of the *B. cereus* group, environmental and intracellular stress signaling routes are combined into one single protein. This activation mechanism may reflect unique niches in which *B. cereus* group members can reside [37].

## Adaptive stress responses and transcriptional landscapes

**Figure 2:**
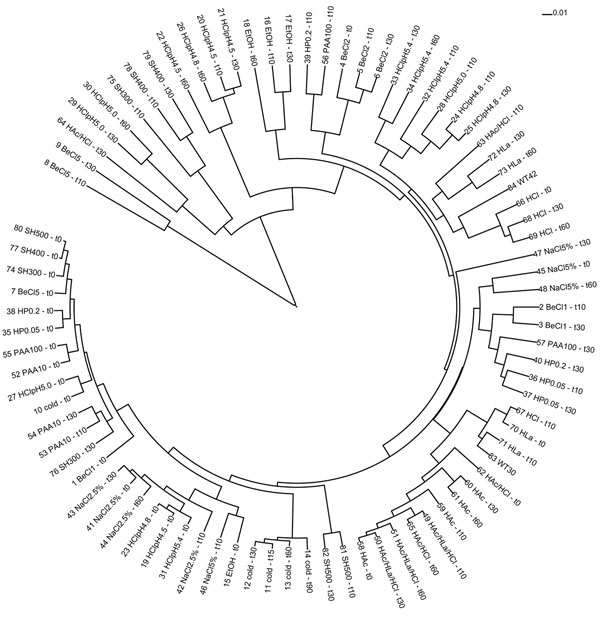
**Neighbour Joining tree of the transcriptome samples.** Samples were arbitrarily numbered 1-84. Time point of sampling is indicated by t<number> in minutes after transfer of the cells to the stress medium. Explanation of the sample description: BeCl; benzalkonium chloride, SH; sodium hypochlorite, PAA; peracetic acid, HP; hydroperoxide, EtOH; ethanol; HCl; hydrochloric acid, HLa; lactic acid, HAc; acetic acid, cold; incubated at 12°C; WT30; incubated at 30^o^C, WT42; incubated at 42^o^C. Unless specifically mentioned in the description, numbers behind the sample description denote mM levels added to the medium. Levels were only indicated if different levels were used for the specific compound across treatments.

The availability of genome-wide transcriptome profiles of *B. cereus* in response to various mild stress conditions including detergents (benzalkonium chloride, hydrogen peroxide, peracetic acid, sodium hypochlorite), ethanol, heat, various acids (HCl, lactic acid, acetic acid; alone and in combinations) and salt stress [42-46] at different time points and concentrations, opens the possibility to identify general as well as specific transcriptional responses of *B. cereus* to these different stress conditions. For comparison of the transcriptomes, the raw data from these different experiments were normalised and scaled using Lowess normalization in Microprep and slide-scaling in Postprep [47]. As the expression profiles of the technical and biological replicates were highly similar, replicate samples were combined to a single expression level per gene (data not shown). Subsequently, the normalized and scaled data were clustered (ad hoc python scripts) and a neighbour joining tree was constructed in neighbor (part of the PHYLIP package) and visualized in iTOL [48]. A clear pattern of organisation of the samples can be observed from this tree (Figure 2). First, most of the control samples (sampled before stress exposure at t=0) clustered at one end of the tree, supporting the idea that the transcriptome profiles of different experimental origin consist of only limited technical bias (e.g. different preculture conditions, microarray batches and bias introduced by different researchers). In addition, the other end of the tree shows the most extreme (lethal) stresses applied to the cells (both the highest time point and (detergent) concentrations). Secondly, the samples in the tree are mainly clustered on the type of stress that was applied and not on time point, suggesting that the response to different stress conditions is the major difference observed within this set of experiments.

**Figure 3:**
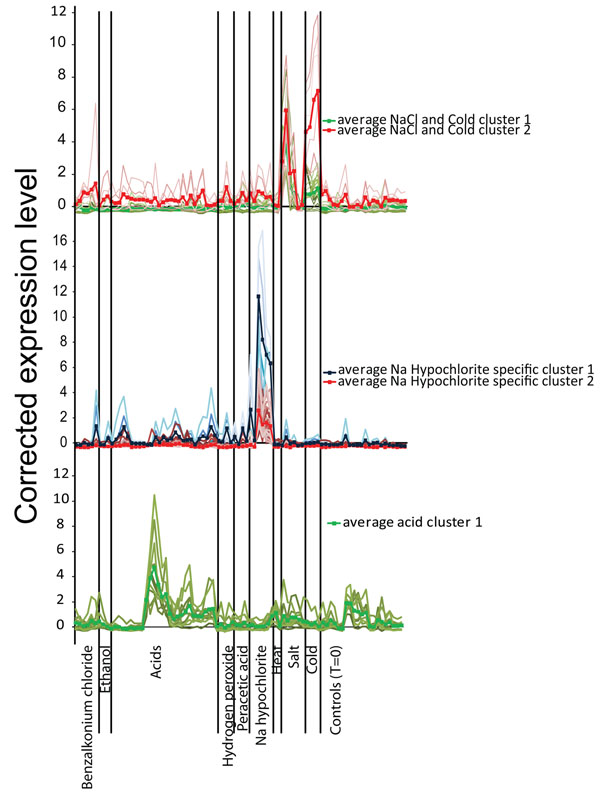
**Transcriptional landscape graphs of three different gene clusters grouped by stress condition.** Different transcriptional landscapes of K-means clustered transcriptome data were obtained and selected landscapes show the (normalized and scaled) expression level (intensity) of all genes (in light colours) within the cluster. In dark colours and indicated by markers for each condition, the average expression level for all genes in the cluster is shown. The three landscapes show the clusters-specific response to sodium hypochlorite (top), to cold stress and salt (NaCl) stress (middle), and acid stress (bottom). The first two landscapes are both consisting of two clusters that differ slightly from each other in the level of activation of gene expression. In the case of the NaCl and cold response landscape this discrimination is associated with the response to cold, for which the response of the second cluster is much higher than that of genes in the first cluster. In case of the sodium hypochlorite landscape the same effect can be observed; the response of genes in the first cluster is much higher than that of genes in the second cluster.

To further asses the specific transcriptome response to different stresses and to determine genes that can be assessed as general stress response biomarkers, the transcriptome data was clustered using K-means clustering (30 clusters, Pearson correlation) in Genesis [49]. These clustering results were manually inspected for clusters with a specific transcriptome response to a single stress or multiple different stresses. Transcriptome landscape figures of these clusters, describing the corrected signal intensity per sample, were constructed and visualized. Several gene clusters were found that respond to specific stress conditions. Three exemplary landscapes for NaCl and low temperature, sodium hypochlorite and acid, are shown in Figure 3. The gene content of these transcriptome landscapes reflect the specificity of the stress response; out of the 22 genes in the clusters specific to cold and NaCl, eight genes are involved in transport of peptides (*BC0684*), salts (e.g. Na^+^, *BC5041*), metals (e.g. cobalt, *BC0160-0162*,) and glycine/proline/betaine (*BC3644*, *BC5238* and *BC3000*). In addition, genes were found encoding for cold shock proteins (*BC2357*) and transcriptional regulators of the CarD family (*BC3648* and *BC4714*). Most of these genes were previously reported by den Besten et al. [43] in their study on transcriptional responses of salt-stressed *B. cereus* ATCC 14579. Notably, a role of proline and betaine, so-called compatible solutes, in growth at low temperature and at high salt concentrations has been reported for a range of bacteria including *L. monocytogenes* and *Bacillus* spp. [2].

The sodium hypochlorite landscape consists of 37 genes in which there is a clear overrepresentation of genes predicted to have reductase activity (*BC1835*, *BC1844*, *BC2194*, *BC3024*,) and arsenate resistance (*BC3152-BC3154*). In addition several transport systems involved in the transport of metal ions (copper, cadmium, zinc) are activated (*BC3731*, *BC3732*, *BC0595*, *BC0596*). Typically, we also observed the activation of the transcriptional regulator *spxA* (*BC3402*) and three MarR-type transcriptional regulators (*BC3025*, *BC4474*, *BC5038*,). These observations extend those of Ceragioli et al. [44], who performed a comparative analysis with cellular responses to three other disinfectants tested, i.e., peracetic acid, benzalkoniumchloride and hydrogen peroxide, and concluded that exposure to sodium hypochlorite induced genes involved in metabolism of sulfur and sulfur-containing amino acids. These results correlated with the excessive oxidation of sulfhydryl groups observed in sodium hypochlorite-stressed cells. For the genes found in the acid cluster it is less clear what their role could be in the response to the stress; apart from three genes coding for proteins with a putative function (*BC2087*, *BC2858*, *BC4640*), some single genes were found to play role in quite diverse processes such as hydrolysis (*BC3414*), phospholipid metabolism (lipase; *BC4333*), iron uptake (*BC5381*), drug resistance (*BC0860*) and peptide breakdown (*BC0789*). Notably, expression activation of genes with putative functions in lipid turnover, iron metabolism and drug resistance have previously been reported for sorbic acid-stressed cells of *B. subtilis*[*50*].

In contrast to the clusters that show clear response to only a single stress, other clusters were found that displayed a more diverse response. One of these clusters contained a large fraction of the σ^B^ regulon, including *sigB*, *rsbW*, an anti-sigma factor antagonist (*BC1002*), *BC099*, and *katE*, and several involved in stress response and DNA repair (*BC0062*, *BC0103*). Another cluster included four genes of the CtsR-regulon (*BC0099-0102*); genes known to code for proteins involved in the repair of protein damage [51]. Other stress response genes were present in clusters of genes active under many different stress conditions, e.g., *hrcA*, *spxA*, *groES/EL*, *grpE*, *clpX* and *BC0377* (putative alkyl hydroperoxide reductase) (data not shown). This pointed to a significant role of these components in general stress adaptation and, as expected, they have known canonical roles in stress adaptation including regulation of stress responses, removing reactive oxygen species, and protein repair and protein quality maintenance [2,51,52]. Although the transcriptome response of the genes in the clusters seems to be very broad, the genes do not respond to all stresses. Especially for the *sigB* cluster, there is a clear distinction between stress conditions under which gene expression is present (benzalkonium chloride, peracetic acid, sodium hypochlorite, heat and salt) and conditions where gene expression was not observed at a high level (cold and hydrogen peroxide). Upon acid stress, the response is even variable between different conditions (depending on the type of acid and the time of exposure). It will be of interest now to find the common denominator among the stresses that trigger σ^B^ as this may point to the exact stimulus/stimuli to which RsbK responds.

## In search of molecular biomarkers for predicting stress-induced bacterial robustness

**Figure 4:**
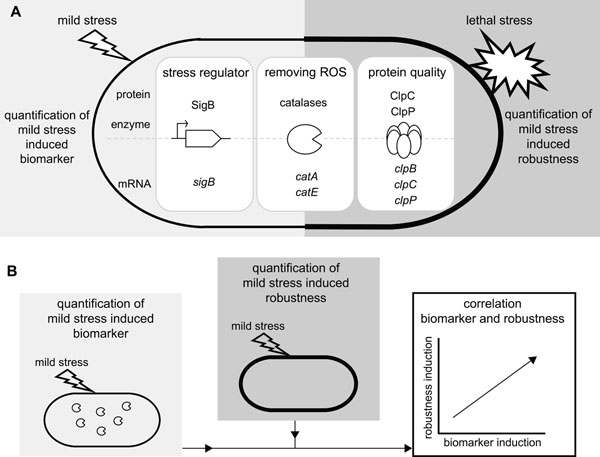
**Conceptual framework for measuring and correlating mild stress induced biomarker and robustness responses and evaluating the predictive potential of biomarkers.** A) Candidate-biomarkers – σ^B^ (activating general stress responses), catalases (removing reactive oxygen species, ROS), and chaperones and proteases (maintenance of protein quality) – were quantitatively measured at transcript, protein and/or activity level upon mild stress treatment. Mild stressed cells were also exposed to lethal stress to quantify their robustness advantage compared to unstressed cells. B) To evaluate the predictive potential of the candidate-biomarker, the mild stress induced biomarker responses were correlated to the robustness advantage of mild stressed cells, and the correlation was tested for significance (figure is adapted from Besten et al. [53]).

The availability of genome-wide transcriptome profiles of *B. cereus* in response to various mild stress-induced conditions including mild oxidative, heat, acid and salt stress [42-45] opened avenues to perform an unbiased search for general stress indicators that could function as biomarkers for mild stress adaptive behavior. The comparison of the mild stress induced transcriptome profiles after 10 min of a mild stress treatment, revealed that only a limited number of genes were differentially up-regulated upon exposure to the selected mild stresses (see above, and [53]). This transcription signature of stress adaptation seemed to be mild stress-independent and directed to potential biomarkers for mild stress adaptation. Several candidate-biomarkers were selected which have known canonical roles in stress responses, namely the transcriptional regulator σ^B^ (activating general stress responses), catalases (removing reactive oxygen species), and chaperones and proteases (maintaining protein quality) (Fig. 4A) [51,52,54]. A framework was designed to evaluate whether these candidate-biomarkers could predict the robustness advantage elicited by mild stress treatment (Fig. 4B). The candidate-biomarkers were quantitatively measured at the transcript, protein and/or activity level upon exposure to mild oxidative, heat, acid and salt stress for various stress adaptation time intervals. Mild stress-treated cells were also exposed to lethal stress conditions (severe oxidative, heat and acid stress) to quantify their robustness advantage compared to unstressed cells. To assess whether the selected candidate-biomarkers — the proteins SigB, ClpC and ClpP, the transcripts *sigB*, *clpB*, *clpC*, *clpP*, *catA* and *catE*, and catalase enzyme activity — could indeed predict the robustness level of mild stress-adapted cells and therefore could function as biomarkers, their induction upon mild stress treatment was correlated to mild stress-induced robustness towards lethal stress (Fig. 4B).

**Figure 5:**
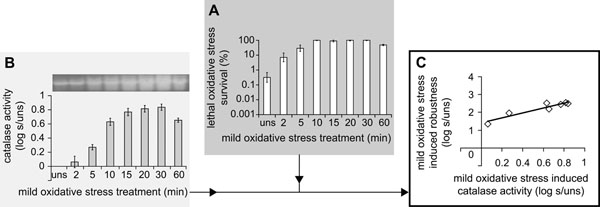
**Catalase activity as potential biomarker for mild stress induced robustness.** A) Unstressed *B. cereus* ATCC 14579 cells (uns) and mild oxidative stress treated cells (0.1 mM H_2_O_2_ treatment for 2 to 60 min) were exposed to 0.2 mM H_2_O_2_ at 30°C for 2 min to determine their robustness towards lethal oxidative stress. The columns mark the number of cells surviving the lethal treatment compared to the initial number of cells (%). B) Catalase activity was measured in unstressed cells and 0.1 mM H_2_O_2_-treated cells. The columns mark the induction of catalase activity after mild oxidative treatment (s) compared to unstressed cells (uns). C) The induction of catalase activity upon mild oxidative stress treatment was correlated to the induction of robustness towards lethal oxidative stress and their correlation was tested for significance. Robustness was determined as the number of cells surviving the lethal treatment, (*N*(*t*)), compared to the initial number of cells, (*N*(*0*)), and the robustness of mild oxidative stress treated cells was expressed relatively to that of unstressed cells, namely, .

Only mild oxidative stress treatment provided (cross-)protection to all three lethal stresses tested, namely towards lethal oxidative stress (Fig. 5A), lethal heat and lethal acid stress [53]. In order to determine whether one or more of the selected candidate-biomarkers could predict these mild oxidative stress induced adaptive traits, the response of the individual candidate-biomarkers upon mild oxidative stress treatment were correlated to mild oxidative stress-induced robustness towards lethal oxidative, heat and acid stress, and the Pearson correlation was tested for significance (*P* < 0.05). As expected, mild oxidative stress treatment highly induced catalase activity (Fig. 5B). Subsequently, this induction pattern of catalase activity upon mild oxidative stress treatment was correlated to the robustness advantage of mild oxidative stress treated cells compared to unstressed cells (Fig. 5C). A stepwise statistical evaluation of this correlation revealed that the induction pattern of catalase activity upon mild oxidative stress treatment was significantly correlated to mild oxidative stress induced robustness towards lethal oxidative stress (Fig. 5C) [53]. This indicated that catalase activity was suitable to predict the oxidative stress robustness enhancement elicited by mild stress pretreatment. Remarkably, catalase activity also emerged as a biomarker for mild oxidative stress induced robustness towards lethal heat stress, as the induction of catalase activity was significantly correlated to the induction of robustness towards lethal heat stress following mild oxidative stress pretreatment [53]. However, the potential of catalase activity to predict the robustness advantage towards lethal acid stress seemed to be non-significant, but only just so. This observation underlined that the predictive quality of selected candidate-biomarkers was also stress-dependent.

In order to evaluate whether catalase activity could also predict the robustness enhancement towards multiple lethal stresses upon pretreatment to mild oxidative stress, the conditions where catalase activity functioned as biomarker, namely lethal oxidative stress and lethal heat stress were combined. The obtained correlation was tested for significance. Because the correlation remained significant also after combining these responses, the results highlight that catalase activity could also act as biomarker for mild stress induced robustness towards multiple lethal stresses [53]. Mild oxidative stress treatment provided cross-protection towards multiple lethal stresses, e.g. lethal oxidative, heat and acid stress. The induction of catalase activity and activation of other stress-related responses upon mild oxidative treatment may have contributed to these multiple cross-protective effects. Recently, it was demonstrated that aerobic lethal heat and acid stress exposure imposed an oxidative stress burden in *B. cereus* cells [55,56], and likely, mild oxidative stress pre-treatment provides a survival advantage for the cells.

The predictive potential of the candidate-biomarkers was highly influenced by the functional cell levels at which the candidate-biomarkers were measured. The *katA* transcript of the main vegetative catalase and the *catE* transcript were both not suitable to predict the robustness status of mild oxidative stress adapted cells towards the three lethal stresses tested [53]. This underlined the importance to evaluate the predictive potential of cellular indicators and different functional cell levels. The better predictive potential of enzyme activity compared to transcript levels might reflect the more transient nature of gene expression than of enzyme activity level. None of the other nine selected candidate-biomarkers could predict the robustness advantage of mild oxidative stress-treated cells, but they emerged as biomarkers for cells that were adapted to mild heat, acid and/or salt. The SigB protein proved to be a suitable biomarker for mild stress-induced robustness towards lethal heat stress, whereas the proteases transcripts *clpC* and *clpP* could predict the mild stress-induced robustness advantage towards lethal acid stress [53].

Catalases have known crucial roles in adaptive stress responses and are widely conserved among bacteria and eukarya [52,57]. The significant role of oxidative stress defense mechanisms in stress adaptation suggests that the predictive potential of oxidative stress related candidate-biomarkers may extend beyond the species *B. cereus*. Moreover, the other selected candidate-biomarkers that have known functions in regulation of stress adaptation and maintenance of protein quality proved to be suitable to predict the robustness advantage of *B. cereus* for various stress adaptive traits [53]. Regulators of general stress responses and cellular systems involved in oxidative stress defense and maintenance of protein quality are among the most consistently induced components in microbial stress responses. Induction of these stress responses upon stress treatment has been demonstrated for a wide range of other micro-organisms, including yeast [58], spoilage organisms [59] and, recently, also for bifidobacteria [60] which are applied in functional foods. Induction of robustness must be averted for food-borne pathogens, but can give organisms used in functional foods a survival advantage. The canonical role of the evaluated stress response systems may point to possible predictive potential also for micro-organisms other than *B. cereus*. Variations in conservation of these components and their regulatory networks underline the need for profound validation of the predictive quality of promising candidate-biomarkers because that might be species or even strain specific. This is clearly illustrated by the regulation of *katA* in *B. cereus* as compared to that in other species. *B. cereuskatA* seems to be negatively controlled by the regulator PerR, just as is the case in *L. monocytogenes*, *S. aureus*, and *B. subtilis*, [*33*]. However, *katA* of *B. cereus* also seems to be (weakly) positively controlled by σ^B^ under stress conditions [32]: a regulatory module/connection that has also been found in *S. aureus*[39,61], but does not seem to occur in e.g. *B. subtilis* and *L. monocytogenes*[54]. In turn, the activation mechanisms of σ^B^ in *B. cereus* differs from that in *S. aureus*, *B. subtilis*, and *L. monocytogenes* i.e., σ^B^ is activated via RsbK-RsbY in *B. cereus* (Fig. 1) [32,37], via RsbU (and putative upstream components) in *S. aureus*[62], and via RsbRSTU in *B. subtilis* and *L. monocytogenes*. In addition, *B. subtilis* uses the RsbPQ pathway to control σ^B^ activity [54].

Our systematic and quantitative approach to search for promising candidate-biomarkers and to evaluate their predictive potential provides perspectives to search for biomarkers in other micro-organisms. The search for biomarkers for stress adaptive behavior will contribute to a better understanding of stress adaptation mechanisms and can serve to detect and predict stress adaptive traits at an early stage.

## Conclusion

In this review, we have integrated three different research strategies that have led to the identification of biomarkers important for robustness in *Bacillus cereus*. Firstly, a general overview of the TCS arsenal of *B. cereus* was obtained using comparative genomics. From this global analysis, a potential key TCS protein (RsbK) could be linked to the earlier defined RsbY-SigB stress-responsive network and further molecular work indeed confirmed the role of RsbK in controlling SigB activity and its regulon. Secondly, a meta-transcriptome data analysis again showed, amongst others, the importance of the RsbKY-controlled SigB regulon in the general *B. cereus* stress response. Thirdly, it was shown that SigB protein levels, protease transcript levels and catalase activity could predict the robustness level of stress-adapted *B. cereus* cells towards lethal stresses.

The drive to use more mechanism-based approaches for evaluating robustness of micro-organisms urged the search for biomarkers to early detect and predict stress adaptive behavior. Prediction of phenotypic behavior using cellular indicators is a key area of research in the field of food-borne pathogens, spoilage organisms, and organisms used in functional food applications [60,63-65]. Quantitatively correlating microbial responses at molecular and phenotypic levels can provide mechanistic understanding of stress adaptive behavior and leads for identifying cellular indicators for bacterial performance. Next to the general stress response regulator sigmaB, cellular components such as catalase activity and proteases were demonstrated to be suitable to predict the robustness level of stress-adapted *B. cereus* cells towards lethal stresses. The predictive quality of the biomarkers was influenced by the functional cell level at which the biomarker was measured, i.e., transcript, protein or enzyme activity level. The level at which the candidate-biomarkers could be employed as biomarker was demonstrated to be stress-dependent. Not only protein levels and enzyme activity but also transcript levels showed high predictive quality. However, the transient nature of expression of transcripts might point to a better predictive potential for the corresponding biomarker if it is assessed at protein and/or enzyme activity level as shown for sigmaB levels and catalase activity. This underlined that evaluation of predictive potential at different functional cell levels is crucial to select robust biomarkers.

Obviously, the different research strategies that were employed may reveal or have already revealed other potential biomarkers for *B. cereus* robustness behaviour, besides the ones mentioned above. For instance, when considering the large arsenal of *B. cereus* TCS proteins, it is noteworthy to mention that of the 51 different HK-RR pairs found in the *B. cereus* group, around 50% was found to be homologous to HK-RR pairs with a known function in at least some form of (general) stress responsive behavior in other species [30,31]. In addition, the biological role for around 20% of the *B. cereus* HK-RR pairs remains completely unknown and it is plausible that at least some of these modules are involved in general stress responsive behavior of *B. cereus*. Finally, 14 different “orphan” HKs are likely to function in sporulation initiation in the *B. cereus* group (Fig. 1) and these may turn out to provide excellent biomarkers for another type of *B. cereus* robustness behavior, namely the formation of dormant endospores.

Further investigation along these lines by varying stress intensities and testing other micro-organisms will reveal how vigorous these quantified correlations are and is required to understand the limits of reliability of the predictive potential of biomarkers. Recently, the response of *Bifidobacterium breve*, a Gram-positive bacterium isolated from the nursling stool of a breast-fed infant, to several stresses (heat, osmotic, solvent, oxidative) was assessed using transcriptomics. Integration of the results with *in silico* analysis, allowed the formulation of a model for an interacting regulatory network for stress response in this bifidobacterium [60], involving HspR, ClgR and HrcA. The latter regulator, and other stress-induced genes in all tested conditions belonging to oxidative stress response and protective proteins such as molecular chaperones and proteases were also generally up-regulated in stressed *B. cereus*[53] suggesting that at least partial overlap in putative biomarkers for robustness may exist. This is further supported by transcriptomic and phenotypic analysis of stress adaptive responses in a range of gram-positive bacteria including lactic acid bacteria [66,67], Bacillus spp. [68,69], and food-borne human pathogens such as *L. monocytogenes*[70,71], that revealed activation of (homologues of ) the proposed canonical biomarkers under a variety of stress conditions.

Studies could be extended with assessment of biomarkers for host-microbe interactions including virulence [72]. Life cycle transcriptomics and comparative genomics have been used recently to unravel successive and coordinated global transcriptional changes during infection of amongst others *L. monocytogenes*, and pointed to previously unknown mechanisms in bacteria with a crucial role for σ^B^-mediated activation of virulence genes in the host intestinal lumen [73].

Robust biomarkers will be of indisputable significance to better understand the mechanisms of stress adaptation, and will complement an empirical approach to evaluate stress robustness of micro-organisms. Insights obtained may on the one hand aid in the design and development of more efficient (novel combination) treatments for control of spoilage organisms and food-borne pathogens and on the other hand provide tools to enhance robustness of organisms used in functional food applications.

## Authors' contributions

MdB carried out the studies on the two component systems and sigmaB, HdB carried out the biomarker studies, MW carried out the in silico transcriptional landscape analysis, TA participated in the design of the studies and its coordination, and all contributed to drafting the manuscript. All authors read and approved the final manuscript.

## Competing interests

The authors declare that they have no competing interests.
